# Benchmark of software tools for prokaryotic chromosomal interaction domain identification

**DOI:** 10.1093/bioinformatics/btaa555

**Published:** 2020-08-27

**Authors:** Mikhail D Magnitov, Veronika S Kuznetsova, Sergey V Ulianov, Sergey V Razin, Alexander V Tyakht

**Affiliations:** Center for Precision Genome Editing and Genetic Technologies for Biomedicine; Group of Genome Spatial Organization, Institute of Gene Biology, Russian Academy of Sciences, Moscow 119334, Russia; Department of Biological and Medical Physics, Moscow Institute of Physics and Technology (National Research University), Dolgoprudny 141700, Russia; Department of Biological and Medical Physics, Moscow Institute of Physics and Technology (National Research University), Dolgoprudny 141700, Russia; Group of Bioinformatics; Laboratory of Structural and Functional Organization of Chromosomes, Institute of Gene Biology, Russian Academy of Sciences, Moscow 119334, Russia; Department of Biology, Moscow State University, Moscow 119234, Russia; Laboratory of Structural and Functional Organization of Chromosomes, Institute of Gene Biology, Russian Academy of Sciences, Moscow 119334, Russia; Department of Biology, Moscow State University, Moscow 119234, Russia; Center for Precision Genome Editing and Genetic Technologies for Biomedicine; Group of Bioinformatics

## Abstract

**Motivation:**

The application of genome-wide chromosome conformation capture (3C) methods to prokaryotes provided insights into the spatial organization of their genomes and identified patterns conserved across the tree of life, such as chromatin compartments and contact domains. Prokaryotic genomes vary in GC content and the density of restriction sites along the chromosome, suggesting that these properties should be considered when planning experiments and choosing appropriate software for data processing. Diverse algorithms are available for the analysis of eukaryotic chromatin contact maps, but their potential application to prokaryotic data has not yet been evaluated.

**Results:**

Here, we present a comparative analysis of domain calling algorithms using available single-microbe experimental data. We evaluated the algorithms’ intra-dataset reproducibility, concordance with other tools and sensitivity to coverage and resolution of contact maps. Using RNA-seq as an example, we showed how orthogonal biological data can be utilized to validate the reliability and significance of annotated domains. We also suggest that *in silico* simulations of contact maps can be used to choose optimal restriction enzymes and estimate theoretical map resolutions before the experiment. Our results provide guidelines for researchers investigating microbes and microbial communities using high-throughput 3C assays such as Hi-C and 3C-seq.

**Availability and implementation:**

The code of the analysis is available at https://github.com/magnitov/prokaryotic_cids.

**Supplementary information:**

Supplementary data are available at *Bioinformatics* online.

## 1 Introduction

High-throughput quantitative surveys of chromatin spatial interactions provide large volumes of data for assessing the 3D conformation of a genome and elucidating interconnections between a genome’s structural and functional organization. Initially, chromosome conformation capture (3C) methods like Hi-C (Lieberman-Aiden *et al.*, 2009) and 3C-seq (Rodley *et al.*, 2009) were applied to investigate the spatial structure of the genomes of higher eukaryotes such as mammals (Dixon *et al.*, 2012; Rao *et al.*, 2014) and *Drosophila* (Sexton *et al.*, 2012; Ulianov *et al.*, 2016). In this context, 3C methods have revealed several layers of chromatin folding: loops and ordinary contact domains are assembled into megabase- and sub-megabase-sized topologically associated domains (TADs) that non-stochastically interact with each other and partition the entire interphase chromatin into compartments.

In contrast, the 3D organization of prokaryotic genomes is more primitive. Bacterial chromosomes are hierarchically folded into large-scale macrodomains that consist of smaller chromosomal interaction domains (CIDs) resembling eukaryotic TADs (Dame *et al.*, 2020). However, these structures appear to be irregularly distributed along the genome and are controlled by nucleoid-associated proteins (NAPs) that form protein–DNA filaments and bridges and thus facilitate DNA looping (Shen *et al.*, 2019). Although only a limited number of prokaryotes have been investigated in terms of genome spatial structure, there is a prominent variation among the 3D organizational patterns of these organisms’ genomes. While the chromosomes of *Caulobacter crescentus* and *Bacillus subtilis* are folded into clearly defined CIDs generated by highly expressed genes and NAPs (Le *et al.*, 2013, 2016; Marbouty *et al.*, 2015; Wang *et al.*, 2015), the *Escherichia coli* genome contains larger interaction units, termed macrodomains, in addition to CIDs, and notably lacks contacts between the right and left replichores (Cagliero *et al.*, 2013; Lioy *et al.*, 2018). Smaller, though less pronounced, CIDs that include co-regulated genes were identified even in the genome-reduced bacterium *Mycoplasma pneumoniae* (Junier *et al.*, 2016; Trussart *et al.*, 2017). *Vibrio cholerae*, a bacterium that carries two circular chromosomes, demonstrates CIDs with structures similar to *C.crescentus* and *B.subtilis*, but also has certain functional trans-contacts between its two chromosomes (Val *et al.*, 2016). Besides the bacteria, the 3D organization of archaea genomes is of particular interest, both because these organisms combine many properties of bacteria and eukaryotes and because the interplay between genome structure and regulation in archaea is of evolutionary interest. In particular, some archaea have histone-like proteins that are involved in the formation of hypernucleosomes (Henneman *et al.*, 2018). A recent pioneering Hi-C analysis of the hyperthermophilic archaea *Sulfolobus islandicus* and *Sulfolobus acidocaldarius* revealed compartment-like structures which had been previously observed only in eukaryotes (Takemata *et al.*, 2019).

Furthermore, Hi-C studies of microbiomes from diverse niches have provided more comprehensive data than conventional metagenomic studies. As demonstrated in a recent study, utilizing Hi-C data along with routine whole-genome ‘shotgun’ sequencing improves the quality of metagenome-assembled genomes (MAGs) to a remarkable extent, which is critical for the detailed description of community composition (DeMaere *et al.*, 2019). The application of 3C methods to the human gut microbiome made it possible to trace the evolution of an entire microbial community in its natural environment, characterize the dynamics of the transfer of genes and mobile elements, and provide evidence of adaptive evolution in core genomes (Yaffe *et al.*, 2020). Moreover, deconvolution of the microbiome Hi-C into contact maps of individual microbes provided a first glimpse into the underexplored diversity of 3D genome organizational patterns (Marbouty *et al.*, 2017).

When state-of-the-art methods are applied to large-scale surveys of the microbiome, which is an extremely complex object in terms of genomic information, it is necessary to evaluate the methods’ precision, efficacy, computational cost and convenience. Such an evaluation can be accomplished through data processing and analysis with simpler single-microbe datasets, similarly to benchmarks that have been performed with eukaryotes (Dali *et al.*, 2017; Forcato *et al.*, 2017; Zufferey *et al.*, 2018). The selection of optimal run parameters and general usage recommendations for these tools is of special interest, as these recommendations might be different as compared to eukaryotic datasets.

In this study, we performed an extensive overview and benchmarking process of most publicly available software tools applicable to microbial CID calling using all available prokaryotic datasets as well as simulated data. Additionally, we propose a new approach for designing a robust Hi-C experiment on prokaryotes based on *in silico* restriction enzyme selection.

## 2 Materials and methods

### 2.1 Sources of experimental data

The following 3C datasets of wild-type bacterial and archaeal species were used for the analysis: *B.subtilis* (Marbouty *et al.*, 2015; Wang *et al.*, 2015), *C.crescentus* (Le *et al.*, 2013), *E.coli* (Lioy *et al.*, 2018), *M.pneumoniae* (Trussart *et al.*, 2017) and *S.acidocaldarius* (Takemata *et al.*, 2019). Of these, only those datasets with at least two biological replicates were used to benchmark domain callers. RNA-seq data for the corresponding species were retrieved from several previous studies (Hess *et al.*, 2013; Le *et al.*, 2016; Vickridge *et al.*, 2017). The datasets’ accession numbers and the reference genomes used are listed in Supplementary Table S1.

### 2.2 Sequencing data processing

#### 2.2.1 Hi-C

Paired-end reads were mapped to the reference genomes using Bowtie v2.2.3 (Langmead *et al.*, 2012) involving the iterative mapping procedure implemented in the *hiclib* package (https://bitbucket.org/mirnylab/hiclib) as described previously (Imakaev *et al.*, 2012). The optimal start position for mapping was determined visually from the read quality profile in FastQC as 4 bp. The minimal read length for mapping was set to 25 bp. The iterative mapping step was increased by 3 bp until a maximum read length was reached. We then filtered out reads that mapped to multiple or zero positions, ‘same fragment’ and ‘dangling end’ read pairs and PCR duplicates. The remaining read pairs were aggregated into genomic bins to produce a Hi-C contact map. For the purposes of benchmarking, we used the bin size described in the original publications for each dataset. The contact maps were iteratively corrected using the ICE algorithm with default parameters, implemented in *cooler* package (Abdennur *et al.*, 2019).

#### 2.2.2 RNA-seq

RNA-seq reads were mapped to the reference microbial genomes using STAR v2.6.1c (Dobin *et al.*, 2013) with an additional –alignIntronMax parameter set to 1 to disable the mapping of spliced reads. Unmapped and low-mapping-quality reads were then removed using SAMtools v1.5 (Li *et al.*, 2009) with option -q 30. To calculate the transcription level, the reads were binned using the same window size as in the corresponding contact maps using BEDtools v2.25.0 (Quinlan *et al.*, 2010). When multiple RNA-seq replicates were available, they were merged after the binning step.

### 2.3 Domain callers

For the comparison, we selected publicly available domain callers based on either scoring function, statistical model, or clustering approaches. Since all domain callers require a pre-processed contact map as input, we applied a common data-processing pipeline, described above, to obtain the contact maps. We then used linear interpolation to fill in missing data to avoid zero-count columns and rows in the contact maps while running the domain-identification algorithms. A CID was defined as a genomic region containing two or more internal bins and two boundary bins. We subjected all the domains annotated by the tools to a filtration step based on their size. For the domain calling procedure, we used the default parameters where possible, except in the case of parameters that depend on the input data and may affect the resulting CIDs. Such parameters were varied to determine the values that maximize the average Jaccard Index (JI) between the domain boundaries across the biological replicates (see ‘Performance metrics’). The description of the parameters and the ranges of variation are given in the Supplementary Tables S2 and S3.

### 2.4 Performance metrics

All analyses were run on Ubuntu 18.04.2 x86_64-linux-gnu on 2 × Intel(R) Xeon(R) CPU E5-2680 @ 2.70 GHz, 192 GB RAM server in a single thread mode. The performance of the tools was assessed using the modified JI (Forcato *et al.*, 2017) and the Measure of Concordance (MoC) (Zufferey *et al.*, 2018). The JI was defined as the ratio of the number of intersecting domain boundaries between two biological replicates and the total number of domain boundaries that the tool identified in both replicates. The MoC was defined as in the original publication (Zufferey *et al.*, 2018). When more than two replicates were available, the metric was calculated across all pairs and averaged. The comparative analysis of the results from this work and previously published benchmarks is given in the Supplementary Table S4.

To assess the biological relevance of annotated CIDs, we used publicly available RNA-seq data. RNA-seq profiles were CPM-normalized and smoothed using the Savitzky–Golay filter with a sliding window of five bins and first-order polynomial approximation. We then extracted the expression levels from ±10 bins around each annotated boundary and calculated the average expression profile at the predicted CID boundaries. Statistical significance was assessed using a Wilcoxon signed-rank test of expression values from five bins around the CID border and five adjacent bins.

### 2.5 Restriction fragments comparison

We performed an *in silico* comparison of the expected restriction fragment sizes produced by various restriction enzymes across the diversity of prokaryotic genomes using the list of representative reference bacterial and archaeal genomes from the NCBI Genome database. Entries with incompletely assembled genomes and with more than one circular chromosome were discarded from the list. The *in silico* genome digestion was performed using the Restriction package from Biopython. For our simulations, we selected four restriction enzymes: HindIII (restriction site: AAGCTT) and HpaII (CCGG), which are the most commonly used enzymes in prokaryotic Hi-C experiments, and NcoI (CCATGG) and MseI (TTAA), which have the opposite GC content of the recognition site.

### 2.6 Hi-C read pairs simulation

To simulate *in silico* Hi-C sequencing, we used the Sim3C software tool (DeMaere *et al.*, 2018). Since Sim3C is unable to predict the real positions of domain boundaries, we used it in a mode with disabled CID simulation. We set the ligation efficiency to 0.75 and the read length to 50 bp. The simulations were carried out for *Clostridium difficile* (NCBI Reference Sequence ID: NC_009089.1), *Bacteroides fragilis* (NC_006347.1), *Bifidobacterium adolescentis* (NC_008618.1) and *Pseudomonas aeruginosa* (NC_002516.2) genomes. For each genome, we generated 15 million paired reads that were analyzed using the data processing pipeline described above to produce a contact map.

### 2.7 Downsampling of contacts

We performed downsampling of the obtained Hi-C matrices using the previously described approach (Yardımcı *et al.*, 2019). The Hi-C matrix was converted into a set of pairwise interactions from which we uniformly sampled a given number of contacts. These downsampled contacts were then re-binned into the Hi-C matrix at a given resolution.

### 2.8 Generation of pseudo-replicates

To evaluate the effect of matrix coverage and resolution on CID annotation, we generated two pseudo-replicates of the *E.coli* dataset. This dataset was selected since it has the highest coverage and therefore could be subsampled into a wide range of total contacts and resolutions. Pseudo-replicates were generated by merging the two biological replicates and then downsampling contacts from the combined matrix.

### 2.9 Coverage and resolution effects

To estimate how the coverage and resolution of contact maps affected the annotation of CIDs by the domain callers, we used the generated pseudo-replicate matrices. We fixed the resolution to 10 kb while testing the effect of coverage (1, 3, 5 and 10 millions of contacts) and the number of total contacts to 20 million while testing the effect of resolution (3, 5, 10 and 15 kb). For all obtained matrices, the domains were identified as described above. To compare the domains obtained for matrices with different coverage, we calculated the number of intersecting boundaries for all pairwise annotations by each tool. To compare the domains obtained for matrices with different resolutions, we allowed an offset of 30 kb for boundary positions and calculated the percentage of overlap between these ‘extended’ boundaries for all pairwise annotations by each tool.

## 3 Results

### 3.1 Genome analysis and simulations can improve experimental design

The restriction enzyme chosen for a chromosome conformation capture experiment defines the resolution limit of the contact map obtained (Pal *et al.*, 2019). According to general practice for eukaryotic genomes, four-base cutter enzymes produce shorter restriction fragments, resulting in higher-resolution contact maps as compared to six-base cutters (Lajoie *et al.*, 2015). While this might also be true for some bacterial species, it may be difficult in many cases to determine whether a four- or six-base cutter would produce a better contact map. For example, when comparing *B.subtilis* contact maps created with HpaII (4-cutter) and HindIII (6-cutter) restriction enzymes (Supplementary Fig. S1A), it is obvious that the four-cutter enzyme outperforms the six-cutter. However, for *C.crescentus* contact maps created with two different six-cutter enzymes, NcoI and BglII, the difference in map quality is also quite dramatic, with NcoI yielding a better contact map (Supplementary Fig. S1B). This variance can be explained by the diversity of prokaryotic genomes with respect to GC content and restriction-site density, suggesting that these parameters should be taken into consideration when selecting a restriction enzyme for the Hi-C/3C-seq library preparation.

**Fig. 1.**
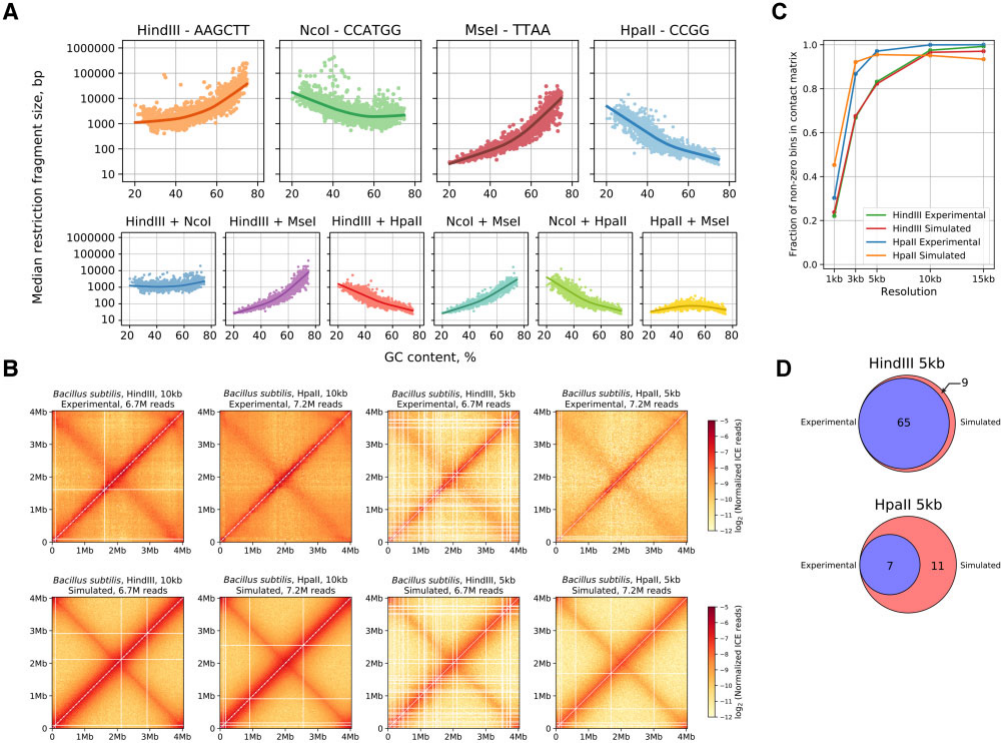
Simulated contact maps reflect the properties of experimental data. (**A**) Scatterplot and LOWESS approximation between genomic GC content and median restriction fragment sizes generated by four restriction enzymes (with varying GC content and recognition-site length) and their combinations. (**B**) Normalized experimental (top row) and simulated (bottom row) Hi-C contact maps for *B.subtilis* generated with HpaII and HindIII restriction enzymes at 10 and 5 kb resolutions. (**C**) The fraction of non-zero cells in experimental and simulated contact maps at different resolutions. (**D**) Venn diagrams showing the overlap of zero-count column and row positions in experimental and simulated contact maps at 5 kb resolution

To analyze how GC content and genome heterogeneity may affect the outcome of a prokaryotic chromosome conformation survey, we collected a representative set of bacterial and archaeal genomes (*N* = 1867) and performed *in silico* digestion experiments. The restriction fragment sizes that directly impact the quality of the contact map were highly dependent on the GC content of the genome (absolute value of Spearman’s *r* > 0.62, *P*-value < 0.001), where enzymes with GC- and AT-richer recognition site sequences more frequently cut genomes with high and low GC content, respectively (Fig. 1A).

To develop a methodology for evaluating whether a restriction enzyme is suitable for an experiment and for estimating an enzyme’s resolution limit and potential contact map quality, we simulated genome digestion and Hi-C sequencing for *B.subtilis* and *C.crescentus* for which real Hi-C data were available. We utilized the restriction enzymes previously used in the Hi-C protocols for these species and downsampled the number of contacts to the same value found in the experimental data (Fig. 1B and Supplementary Fig. S2A). We binned each of the simulated and experimental maps across a range of resolutions (3, 5, 10 and 15 kb) and compared them by calculating the fraction of non-zero cells in the map. These values appeared to be highly correlated (Pearson’s *r* > 0.98, *P*-value < 0.001) between simulated and experimental *B.subtilis* maps (Fig. 1C) for both HpaII and HindIII enzymes. Given a certain number of contacts, this metric can be used to estimate the upper-bound map resolution achievable for a specific genome-enzyme combination. When comparing rows and columns containing only zeros, which represent ‘problematic’ bins with an extremely large or small number of restriction sites and/or low chromatin accessibility, we observed that the experimental maps were reproduced in the simulation at both 5 and 10 kb resolutions (Fig. 1D). Similar results were obtained from the *C.crescentus* simulations with the NcoI and BglII restriction enzymes (Supplementary Fig. S2B and C). Taken together, these results show that our simulation represents the real data quite well and can be used during the design of a prokaryotic Hi-C experiment.

To illustrate the practical relevance of the suggested method, we applied this procedure to several bacterial genomes with widely varying GC content (29–66%) for which the 3D genome structure is yet to be elucidated. As expected, we observed that simulations with restriction enzymes that have theoretically shorter restriction fragments produced much better contact maps. Moreover, there was a clear positive association between bacterial GC content, restriction site GC content and map quality (Supplementary Fig. S3), supporting our hypothesis that it is important to select an optimal restriction enzyme for each prokaryotic species during Hi-C analysis.

### 3.2 Domain annotations vary significantly between the tools

Previous analyses of prokaryotes using Hi-C technology revealed several layers of their 3D architectural organization, such as compartments (Takemata *et al.*, 2019), macrodomains (Lioy *et al.*, 2018) and CIDs (Le *et al.*, 2013). To annotate the latter, it is necessary to have: (i) a high-resolution contact map, typically not less than 15 kb, and (ii) a suitable algorithm for domain calling. One current limitation is that most domain calling algorithms have been developed for eukaryotes, whose domains appear slightly differently on contact maps. We therefore aimed to perform a comprehensive benchmark of the available domain callers to determine their potential application to prokaryotes.

For the comparative analyses, we used experimental 3C data from five prokaryotic studies that had different numbers of samples and involved protocols with different restriction enzymes (Supplementary Table S1). A uniform data processing pipeline was applied to all samples to increase the comparability of the results and facilitate the CID calling procedure. We compared the performance of 29 common domain callers using two metrics: the JI, which measures the normalized overlap between annotated domain boundaries, and the MoC, which calculates the overall silhouette similarity between two CIDs’ segmentations of a genome.

**Fig. 2.**
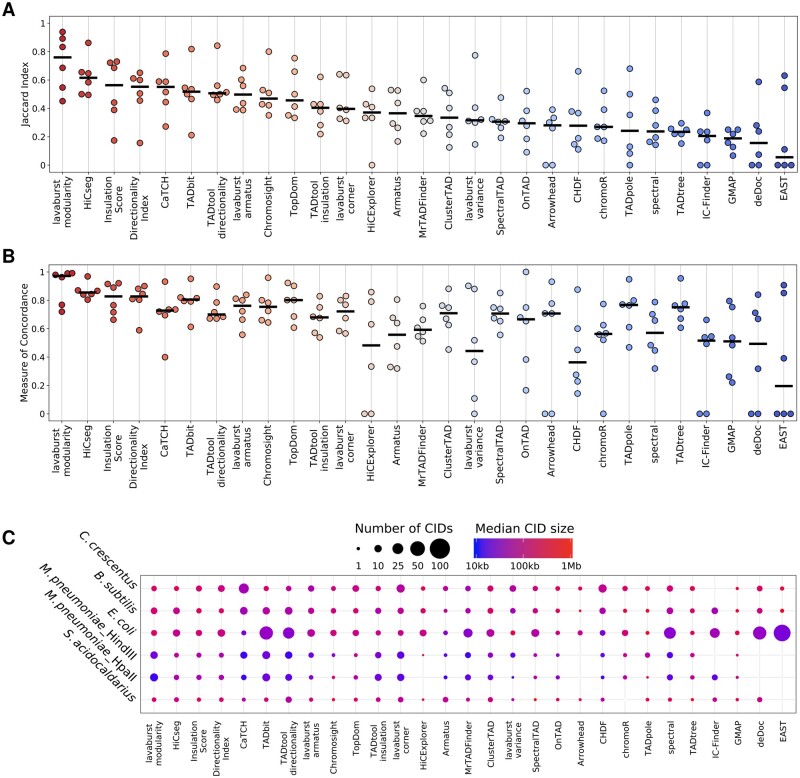
Comparative analysis of annotated domains in prokaryotes. Swarm plots of the JI (**A**) and MoC (**B**) metrics for pairwise comparison of domain segmentations between biological replicates. Tools are ordered by median JI value, from highest to lowest. Each dot represents the average score for a single dataset. Black lines represent the median value for each metric across all datasets. (**C**) Bubble chart for the number of CIDs (represented by bubble size) and their median sizes (represented by colour-scale) annotated by each domain caller for each dataset

Overall, the CIDs’ segmentations between the biological replicates appeared to be rather stable and were identified by all tools (Supplementary Fig. S4); nevertheless, the exact positions of the boundaries remain ambiguously mapped as measured by the JI and MoC metrics (Fig. 2A and B, respectively). The domain-annotation similarities were very high across all tools except HiCExplorer and ClusterTAD (median MoC of 0.71), while the median boundary reproducibility was lower (median JI of 0.35) and only seven tools had JI scores above 0.5.

Across all prokaryotic datasets, lavaburst.modularity, HiCseg, Insulation Score and Directionality Index showed the highest reproducibility of boundaries (JI > 0.55) and the highest concordance between domains (MoC > 0.83) for biological replicates within the same study.

Surprisingly, four out of five tools demonstrating the lowest consistency between boundaries (deDoc, GMAP, IC-Finder and TADtree) had the average level of concordance for the domain positions themselves (Fig. 2A and B). However, the robustness of the boundary annotations was generally superior, since this metric is independent of the number of CIDs, their sizes and their relative positions.

To evaluate the consistency of domain boundaries as identified by different tools for the same dataset, we calculated how frequently each tool’s annotated CID boundaries were identified by other tools (the percentage of shared boundaries; Supplementary Fig. S5A). On average, all tools had comparable proportions of shared boundaries (∼85%), however, most of them tended to be shared with only one to four other tools. Among all tools, IC-Finder, lavaburst.variance and EAST had the highest proportion of unique boundaries—i.e. those that were not detected by any other tool—while TopDom, OnTAD and TADpole had the highest proportion of shared boundaries (>50% were detected by more than five other tools).

We also examined the internal properties of the annotated domains. The number and sizes of the CIDs annotated by different tools varied considerably when compared between biological replicates (Fig. 2C). For example for *E.coli*, EAST annotated on average 225 domains per replicate, while TADpole identified an average of only seven. For all datasets, Arrowhead annotated the fewest CIDs (66 in total), while TADbit annotated the most (704 in total). The median CID sizes for both *C.crescentus* contact maps at a resolution of 10 kb ranged from 40 kb for CaTCH and MrTADFinder to 610 kb for GMAP. Moreover, some tools (GMAP, TADpole and HiCExplorer) consistently annotated larger CIDs than the others.

Notably, while some CID segmentations covered most of the genome (e.g. greater than 90% coverage with lavaburst.modularity, HiCseg, Insulation Score, Directionality Index, TADbit, Chromosight, TopDom, SpectralTAD, chromoR and TADpole), almost one-third of the tools did not produce continuous CID segmentations (e.g. less than 40% coverage with CaTCH, HiCExplorer, Armatus, Arrowhead, CHDF, IC-Finder and EAST), omitting almost half the genome (Supplementary Fig. S5B).

We additionally evaluated the running time of each tool using 5 and 10 kb contact maps (Supplementary Fig. S6). The running time of most tools did not exceed 30 s for a 5 kb and 5 s for a 10 kb contact map, respectively.

### 3.3 Coverage and resolution might affect CID identification performance

To investigate the impact of coverage and resolution on the identified domains, we generated contact maps of pseudo-replicates using a merged *E.coli* dataset for a range of contact numbers and resolutions that represent the actual parameters of the prokaryotic Hi-C experiments.

All tools except Arrowhead, CHDF and spectral showed high agreement of the CID boundaries annotated at different coverages (Supplementary Figs S7 and S8). On the other hand, some but not all of the tools (e.g. lavaburst.corner, lavaburst.modularity and Directionality Index) robustly annotated CIDs across different resolutions (Supplementary Figs S9 and S10). Generally, the concordance of each tool’s CID segmentations was higher across varying contact map coverage depths (median MoC of 0.76) than across varying contact map resolutions (median MoC of 0.46). Lavaburst.modularity and lavaburst.corner exhibited the best performance across coverage depth and resolutions, respectively. Overall, lavaburst.modularity, HiCseg, Insulation Score and Directionality Index demonstrated robust CID detection across the selected set of coverage depths and resolutions, similarly to their performance with the prokaryotic datasets chosen for this study.

**Fig. 3.**
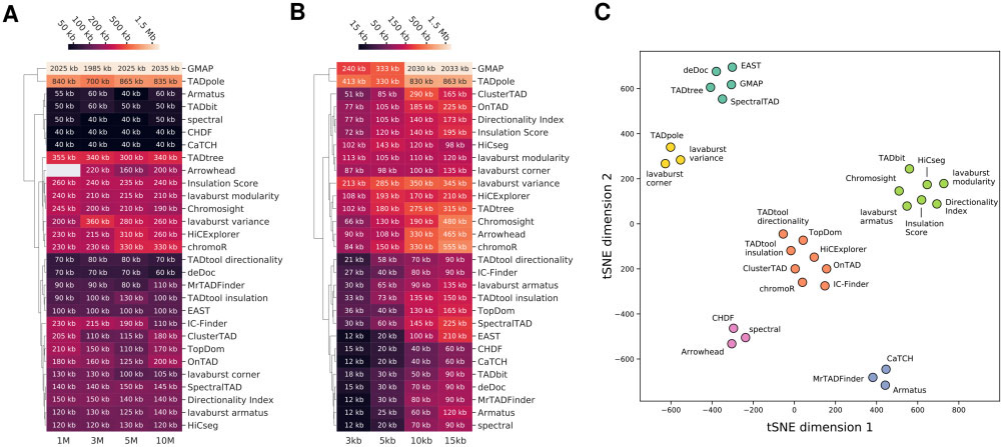
Properties of the domains annotated at different coverage depths and resolutions. Clustered heatmaps of the median size of CIDs annotated in the *E.coli* pseudo-replicates across different (**A**) coverages and (**B**) resolutions. (**C**) Scatter plot of the t-SNE performed on the matrix of MoC values across coverage and resolutions and JI values obtained for the segmentations between biological replicates. Clusters were annotated using the *k*-means clustering algorithm

Domain callers should exhibit consistent annotation of comparably sized CIDs across coverage depths as well as an inverse relationship between CID size and map resolution. Therefore, we categorized all tools according to these criteria by calculating their median annotated domain sizes (Fig. 3A and B). As expected, most tools demonstrated the proper responses to changes in both coverage and resolution. However, the predictions of a number of the tools (HiCExplorer, lavaburst.variance, ClusterTAD, Arrowhead, TADtree and TADpole) failed to exhibit the desired trends.

Overall, the results demonstrate that some of the domain calling algorithms that we benchmarked are insensitive to the coverage and resolution of the contact map (Fig. 3C), which makes them suitable for use on prokaryotic datasets with varying sequencing depths and resolutions.

### 3.4 Validation of identified domains using gene expression data

While the robustness of CID detection across replicates supports the technical relevance of a given algorithm, the concept of CIDs is operational rather than ultimate and therefore requires validation from additional layers of biological information. For a number of bacteria, CID boundaries have been reported to be enriched in highly expressed genes and NAPs (Dame *et al.*, 2020). Therefore, the level of their abundance at CID boundaries, as compared to the regions within CIDs, is a potential measure of the quality of annotated domains.

We further validated the domain annotations produced by the four best-performing tools (lavaburst.modularity, HiCseg, Insulation Score and Directionality Index, as discussed above) by calculating genome-wide expression levels for *C.crescentus*, *B.subtilis* and *E.coli* with available RNA-seq data and comparing expression distribution at and between CID boundaries (Supplementary Figs S11 and S12). This analysis revealed that, surprisingly, lavaburst.modularity, the tool that had the highest JI and MoC scores across all datasets, demonstrated a very poor enrichment of gene expression at the boundaries for *B.subtilis* and *E.coli* (Wilcoxon signed rank test, *P*-value > 0.1). However, Insulation Score and Directionality Index, which did not exhibit the best reproducibility scores, produced results that aligned more closely with expected patterns, showing a statistically significant increase in expression around CID boundaries for all three bacteria when validating domain boundaries with RNA-seq data (Wilcoxon signed rank test, *P*-value < 0.05). This example shows that in some cases, a high concordance between the CID boundaries has nothing in common with the underlying biology, driven by transcription and protein occupancy of DNA, and is only an intrinsic property of the domain caller and the segmentation itself.

## 4 Discussion

Our initial attempt to comparatively analyze domain calling algorithms for use with prokaryotes led to a further effort to optimize experimental designs by selecting the best restriction enzyme for a specific microbe. Notably, variable GC content and restriction site distribution along the genome imply local deteriorations in the quality of Hi-C contact maps, particularly in the vicinity of horizontal gene transfer hotspots in prokaryotes (Ravenhall *et al.*, 2015). To address these issues, one can use a mixture of two or more restriction enzymes to capture more contacts and improve the map quality. Our *in silico* analysis showed that it is possible to use computational methods to accurately predict which restriction enzymes are appropriate for an experiment. This method could be especially useful for experiments that involve microbiome samples containing bacterial species which may have either low or high GC content. Additionally, conducting preliminary profiling of a microbiome composition using cost-effective methods like 16S rRNA sequencing could help to evaluate both community structure and the extent of GC variability across its member taxa. The proper choice of restriction enzyme also has implications for advanced applications of Hi-C to metagenomic data, such as the binning and assembly of MAGs (DeMaere *et al.*, 2019) as well as the identification of microbial hosts for phages and antibiotic-resistance genes (Stalder *et al.*, 2019). While our observations may prove useful for the design of experiments with prokaryotes, they are not the only relevant criteria; other factors like methylation sensitivity and reaction conditions may also be important for the selection of restriction enzymes.

Next, we used a curated list of publicly available wild-type microbial Hi-C datasets to evaluate the domain callers. Bacterial mutants were intentionally excluded from our analysis to avoid confounding effects; however, our recommendations may also facilitate the investigation of chromosome packaging mechanisms in bacteria based on genome-perturbation models. Since data are available for only a single strain per species, we note that CIDs may vary greatly across related strains of the same species. In the future, it would be interesting to explore how domain positions are associated with translocations of genes and operons, polymorphisms and other inter-strain genomic differences. It would also be interesting to investigate how CIDs vary across different environmental conditions (heat or cold shock, osmotic stress, etc.), as well as in different growth phases, since changes in gene expression can dramatically affect bacterial genome conformation and vice versa (Shen *et al.*, 2019; Wolf *et al.*, 1999).

We developed three basic criteria to identify high-quality domain calling algorithms. First, the algorithm should be technically robust, which means that it should produce similar results for two contact maps of experimental replicates. A higher concordance between these contact maps implies a better domain annotation, as supported by our observations from the analyzed pseudo-replicates. Notably, it was at this stage that considerable variability between the tools was detected and superior tools were selected (lavaburst.modularity, HiCseg, Insulation Score and Directionality Index). Second, a good algorithm should yield CIDs that are not dependent on the coverage and resolution of the Hi-C data. Interestingly, most of the evaluated tools succeeded in this respect, which makes them suitable to use for prokaryotic datasets that vary in sequencing depths and resolutions. Furthermore, the tools are also applicable for Hi-C analyses of microbiomes, where the relative abundance of different microbes can vary by orders of magnitude. Third, a good algorithm should produce CIDs that are concordant with the other biological properties of the organism, as reflected by linear genomic features such as gene expression, gene density and operon structures, histone-like proteins and NAPs binding. We utilized RNA-seq data as an example to show how such data can be used to assess the reliability of domain annotation. Our results showed that when selecting a tool, it is important to remember that it might be robust technically but not biologically. In particular, among the four tools that were highly ranked in terms of concordance between replicates and coverage and resolution stability (lavaburst.modularity, HiCseg, Insulation Score and Directionality Index), only two performed well in terms of correlation between CID boundaries and gene expression. To put these results in perspective, it would be intriguing to evaluate the extent of the association of gene expression and CIDs across the diverse branches of the prokaryotic tree of life, and how this association scales with the size of genes and operons.

When comparing the performance of the tools described here with the previously published benchmarks (Supplementary Table S4), we have found that the list of the tools exhibiting high reproducibility between the biological replicates is remarkably the same. However, some algorithms shown to work well for eukaryotic datasets failed to reliably annotate prokaryotic CIDs or were not efficient as measured by other metrics. While comparing the sensitivity to coverage and resolution of the contact maps, the majority of the tools presented here are more robust to coverage than to resolution, similar to the results reported previously. Despite the overall fractions of the shared domain boundaries being generally lower for prokaryotes, the top-scoring algorithms from our list were able to produce the biologically relevant results supported by the gene expression data.

We note that the very concept of prokaryotic CIDs is yet to be validated by observations of a wider range of single microbes. A major hurdle in partitioning bacterial genomes into contact domains is that CID boundaries are not stable; perhaps, more precise descriptions such as insulation profiles or preferential contact distributions should be examined without generalizing to the domain level. Single-cell prokaryotic Hi-C experiments might prove useful here, as they capture alternative 3D genome conformations that are distinct from cell to cell. Apparently, some prokaryotes may not even manifest domain structure, as has been shown in archaea in two *Sulfolobus* species (Takemata *et al.*, 2019). It remains to be explored which phylogenetic branches exhibit defined 3D patterns in their genomic structure, as well which conditions pertain to those patterns.

The applicability of our recommendations might be affected by the many other factors that play a role in 3C experiments, such as the variable efficacy of bacterial genome fixation, ligation and restriction, the thickness of prokaryotic cell walls, etc. However, the general methodological advice and optimal run parameters that we identified for the domain callers that we benchmarked are more general and may facilitate future studies in the 3D genomics of prokaryotes, as well as serve as a reference when performing CID identification for microbes. We anticipate that our analysis will provide guidelines for further investigations of prokaryotic genome organization using high-throughput chromosome conformation capture methods.

## Funding

This work was supported by the Russian Science Foundation [19-74-10092]. M.D.M. and A.V.T. were supported by the Ministry of Science and Higher Education of the Russian Federation [075-15-2019-1661]. S.V.U. and S.V.R. were supported by the Russian Foundation for Basic Research [18-29-07001]. Funding for open access charge: Russian Science Foundation [19-74-10092].

*Conflict of Interest*: none declared.

## Supplementary Material

btaa555_Supplementary_Data
