# Supplementary material for: Benchmark of software tools for prokaryotic chromosomal interaction domain identification
Source: Bioinformatics. 2020 Aug 27;36(17):4560–7. doi: 10.1093/bioinformatics/btaa555 (PMC7653553; doi:10.1093/bioinformatics/btaa555)
Supplement: btaa555_Supplementary_Data [file btaa555_supplementary_data.pdf]

Supplementary Information

Benchmark of software tools for prokaryotic chromosomal interaction domain identification

Magnitov et al.

- Supplementary Figures S1-12
- Supplementary Tables S1-4

Supplementary Figure S1

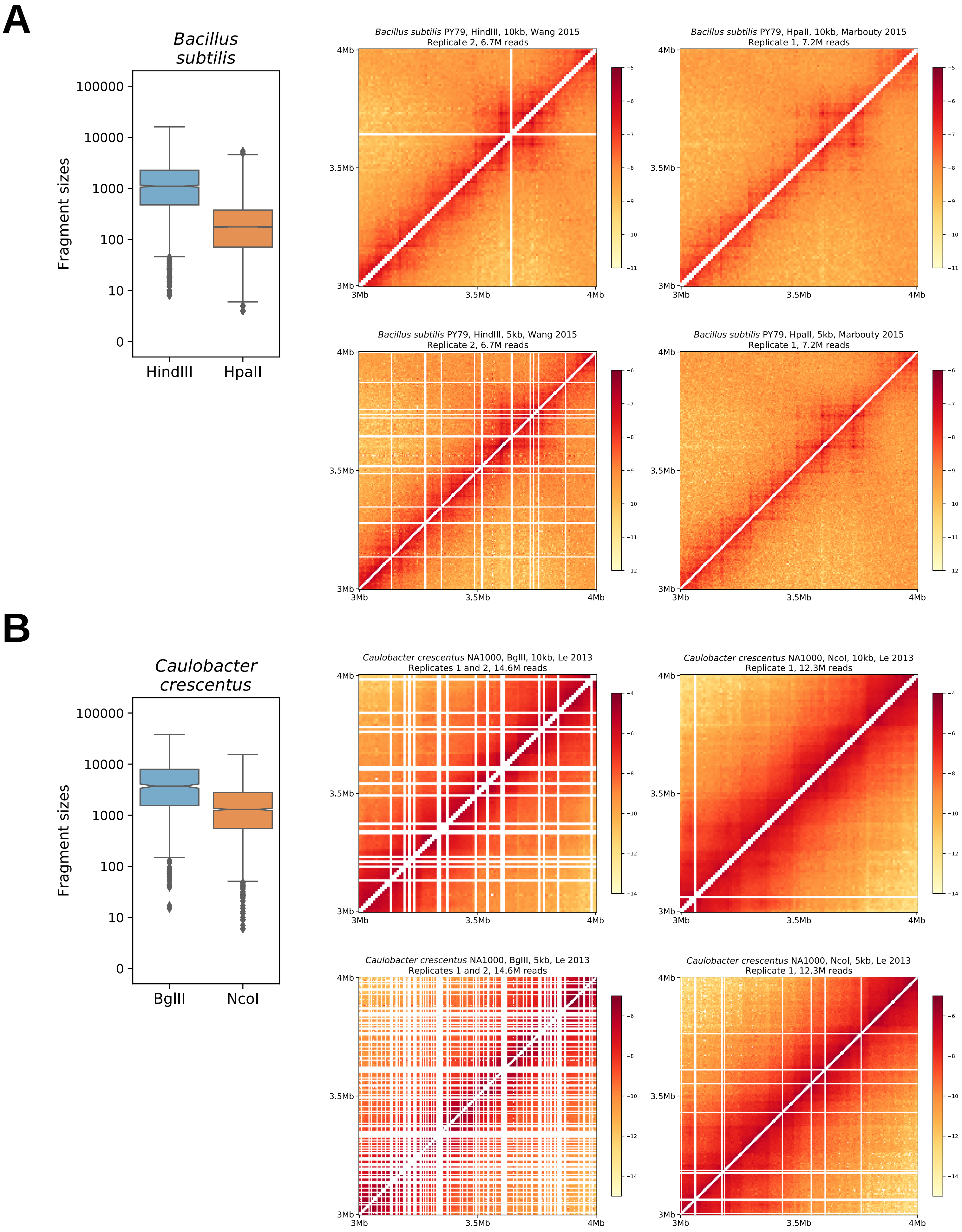

Supplementary Figure S1. Comparison of restriction fragment sizes and the quality of experimental contact maps. (A) Boxplot of the HindIII and HpaII restriction fragment sizes (in base pairs) and contact maps at 5 and 10 kb obtained using the respective restriction enzymes for the *B. subtilis* genome. (B) Boxplot of the BglII and NcoI restriction fragment sizes (in base pairs) and contact maps at 5 and 10 kb obtained using the respective restriction enzymes for the *C. crescentus* genome.

Supplementary Figure S2

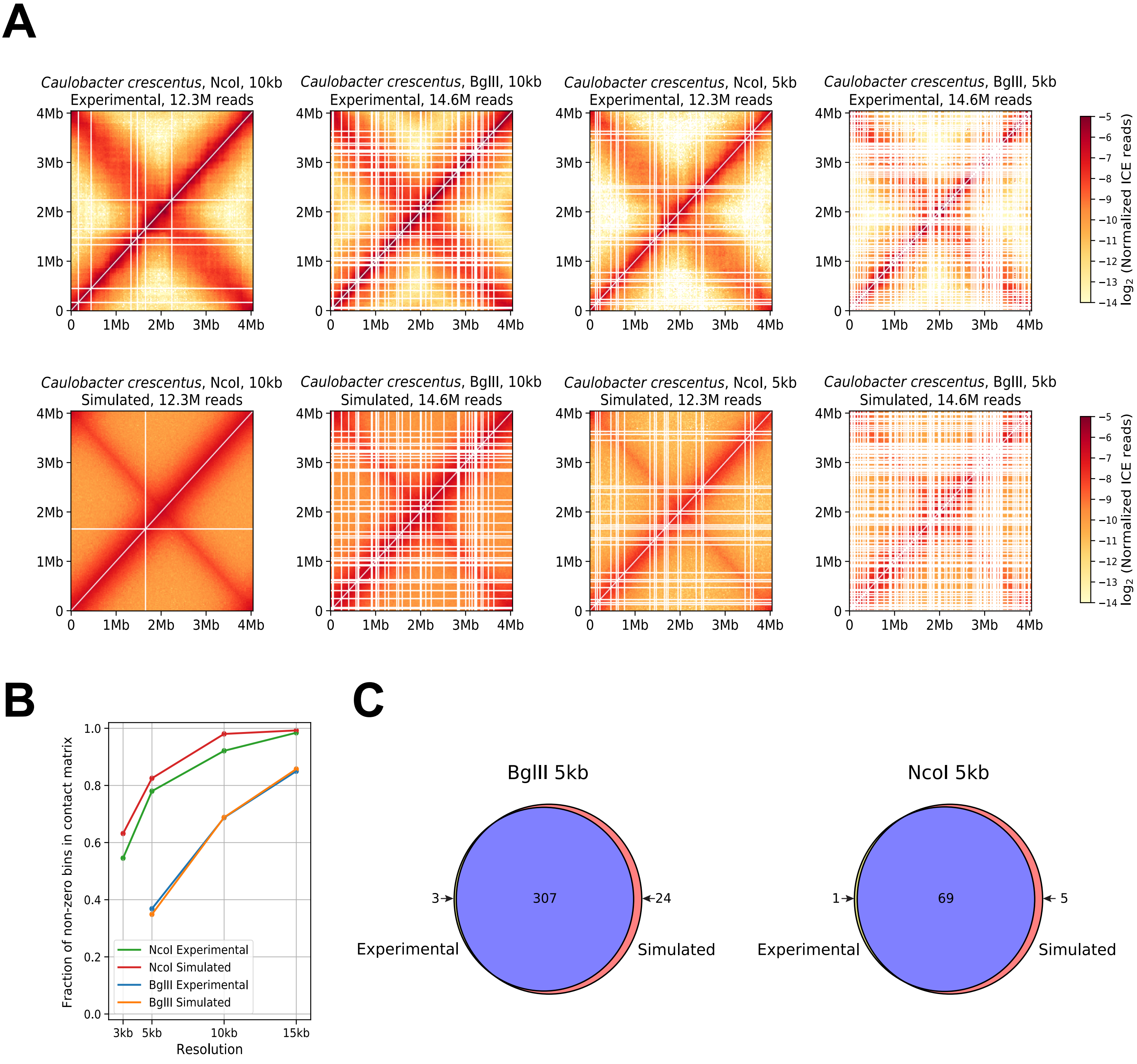

Supplementary Figure S2. (A) Normalized experimental (top row) and simulated (bottom row) Hi-C contact maps for *C. crescentus* generated with NcoI and BglII restriction enzymes at 10 and 5 kb resolutions. (B) The fraction of zeroed cells in experimental and simulated contact maps at different resolutions. (C) Venn diagrams showing the overlap in positions of zero-count columns and rows for experimental and simulated contact maps at 5 kb resolution.

Supplementary Figure S3

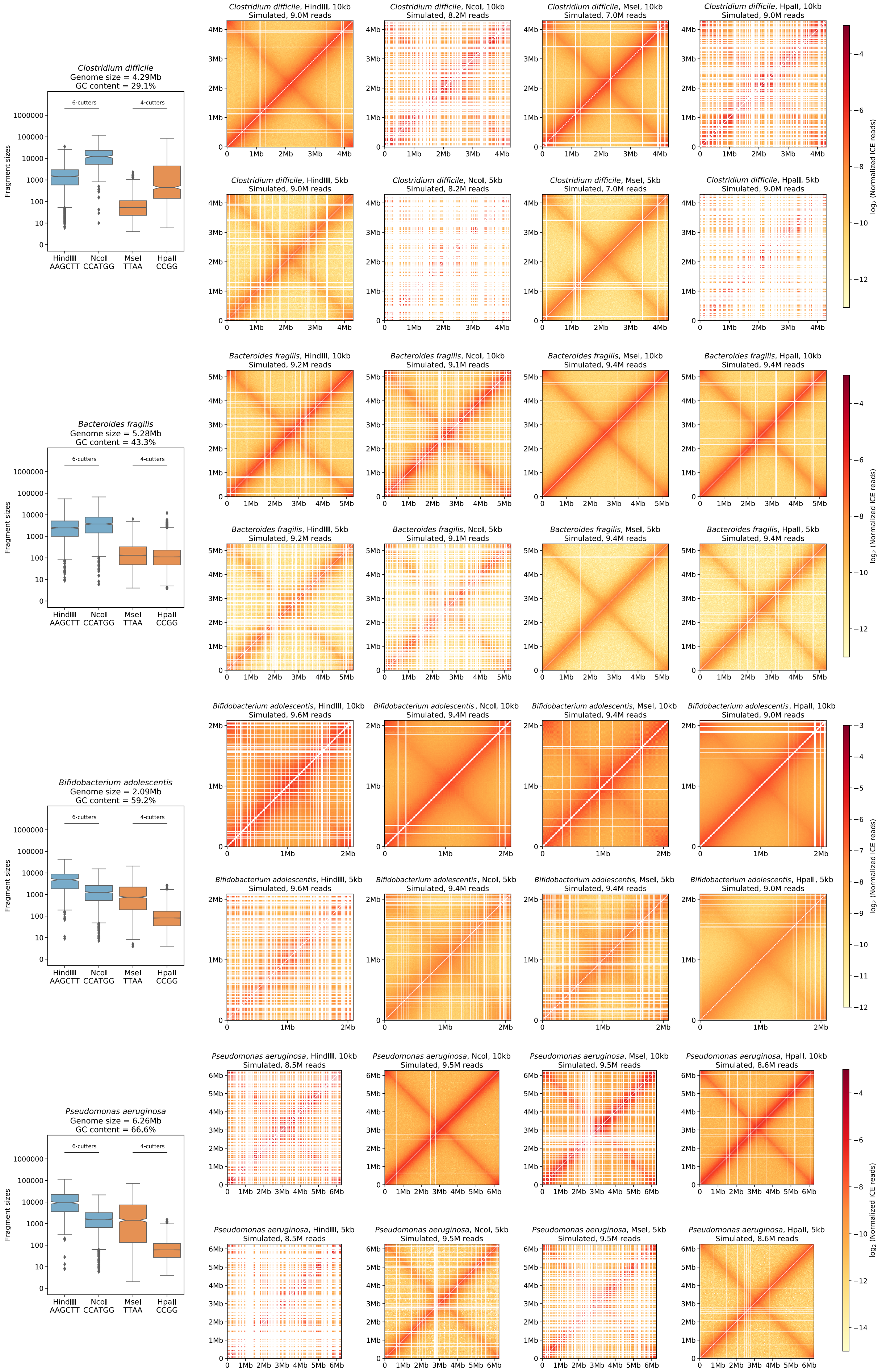

Supplementary Figure S3. Boxplots of the estimated restriction fragment sizes (in base pairs) and simulated contact maps for selected bacterial genomes with varying GC content. Generally, maps simulated with restriction enzymes with theoretically shorter restriction fragments have higher quality. When comparing two 4-cutter enzymes, MseI (TTAA recognition site) yields a better map when the genome has a lower GC content (third row), while HpaII (CCGG) exhibits the opposite trend. Similar results are observed for the HindIII (AAGCTT) and NcoI (CCATGG) restriction enzymes, which are 6-cutters that vary by the GC content of their recognition sites.

Supplementary Figure S4

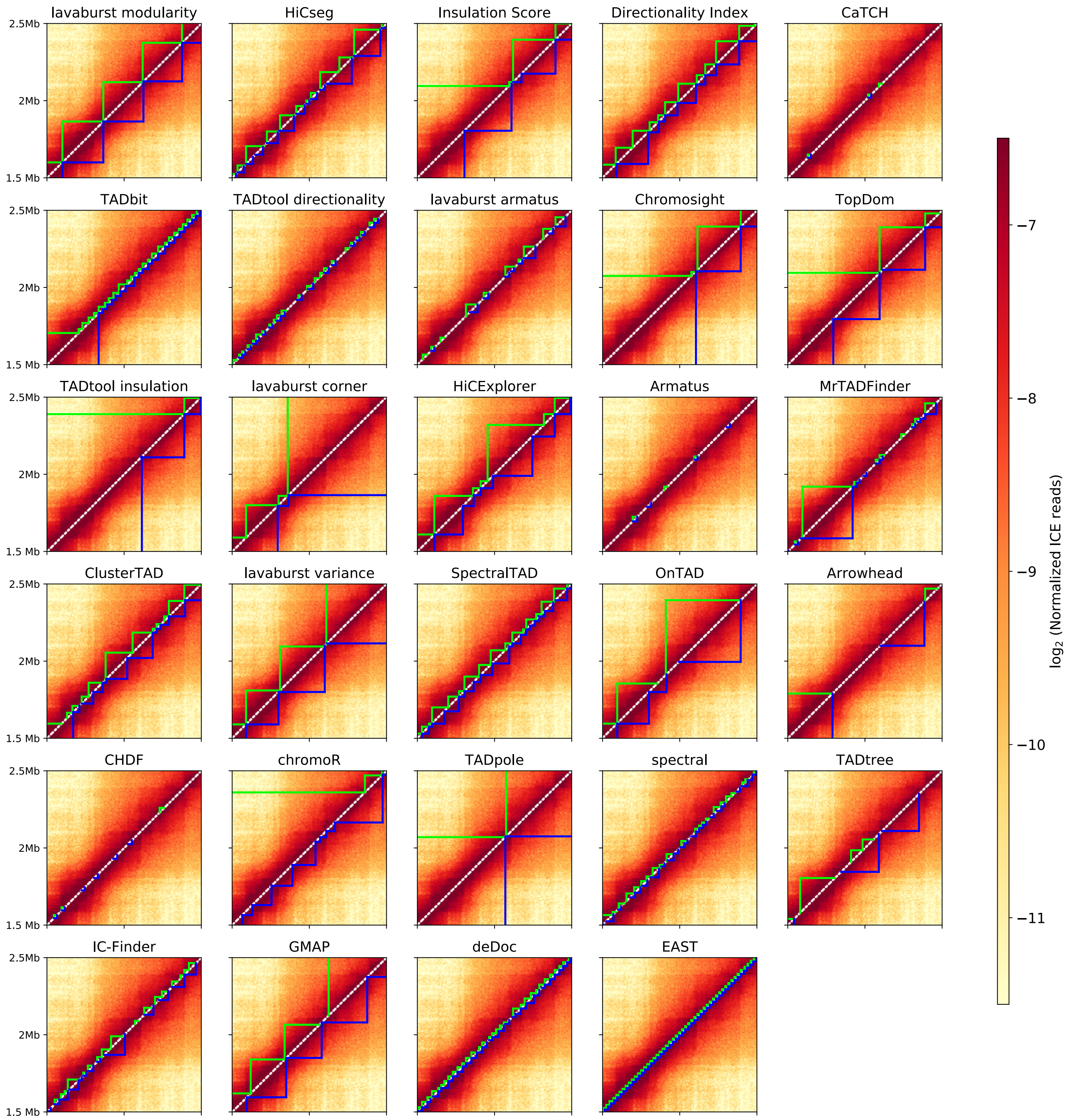

Supplementary Figure S4. Examples of the CIDs annotated for *E. coli* replicates at the 5 kb resolution, including the domains annotated in replicate 1 (in green) and replicate 2 (in blue).

Supplementary Figure S5

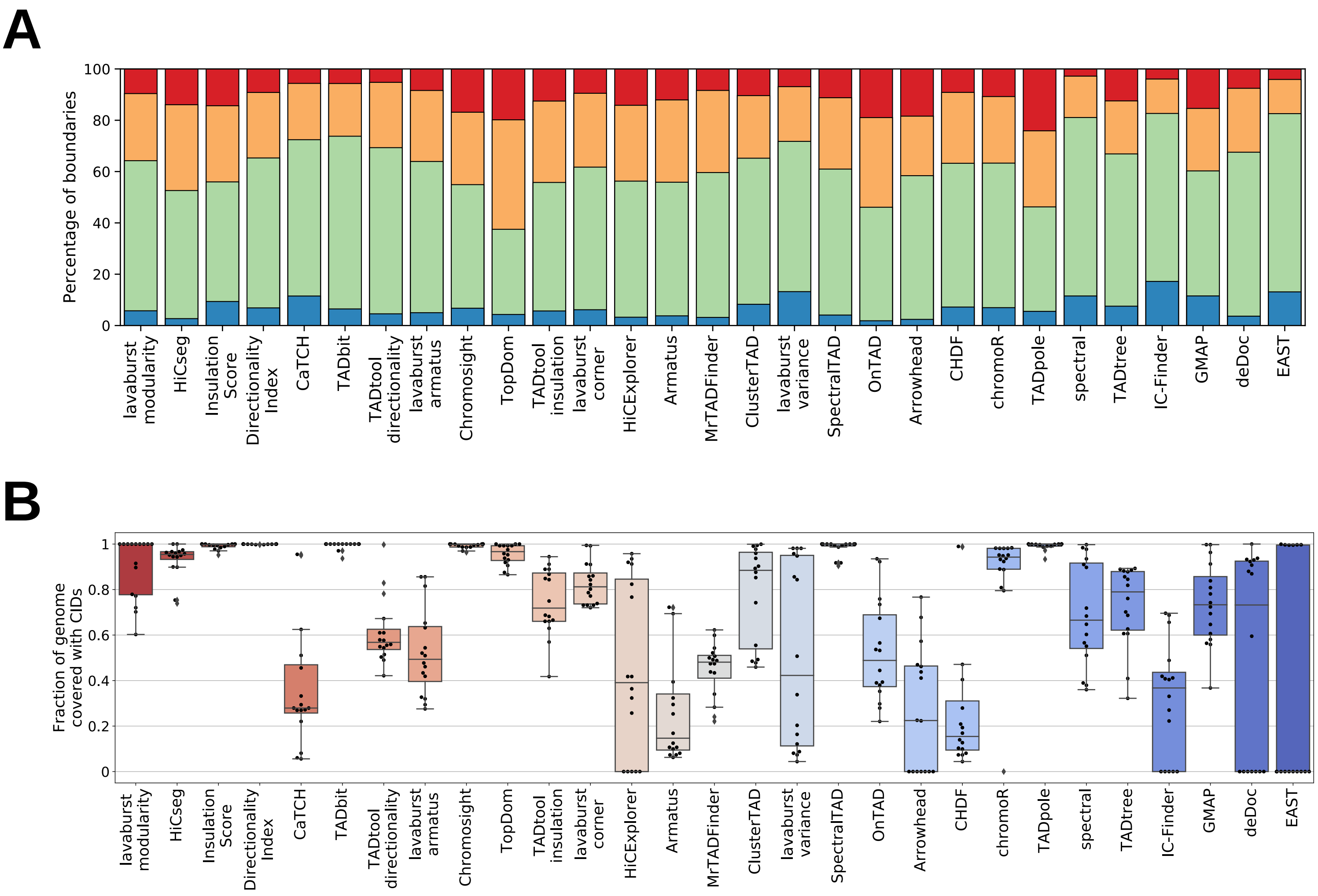

Supplementary Figure S5. (A) Fraction of CID boundaries identified by each domain caller that were also identified by 0 (blue), 1 to 4 (green), 5 to 8 (orange) and 9 or more (red) other tools. (B) Boxplots of the total genome fraction covered by CIDs for all domain callers. Segmentation for each biological replicate used in the benchmark is represented by a dot (N = 16). Tools are ordered by median Jaccard Index value, from highest to lowest.

Supplementary Figure S6

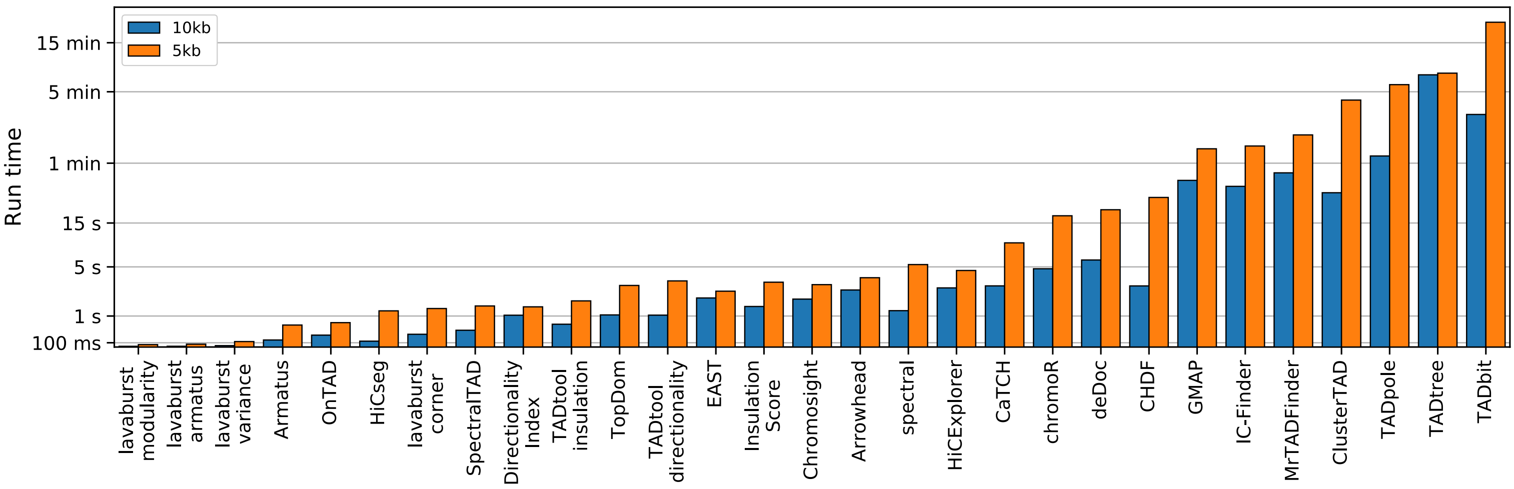

Supplementary Figure S6. Running time of the 29 TAD callers calculated for 5 and 10 kb contact matrices from *E. coli* pseudo-replicates. Tools are ordered by increasing average run time, from highest to lowest (ordered by increasing average value).

Supplementary Figure S7

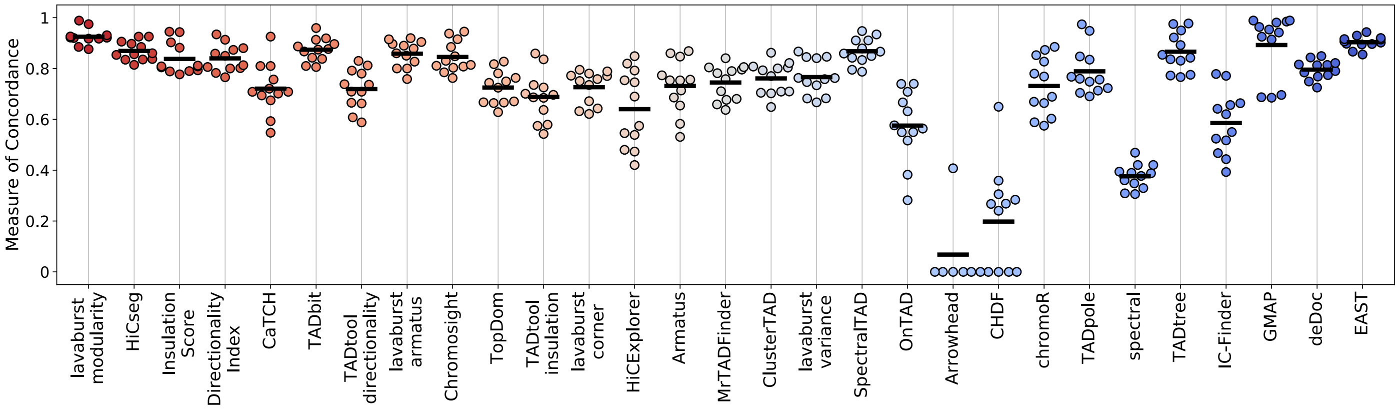

Supplementary Figure S7. Swarm plots for the Measure of Concordance values between the CID segmentations annotated for *E.coli* pseudo-replicate contact maps with varying coverage (1, 3, 5 and 10 millions of contacts) by each domain caller. Each dot represents a comparison between CID annotations obtained for two maps with different numbers of contacts. The black line represents the median MoC value for each tool.

Supplementary Figure S8

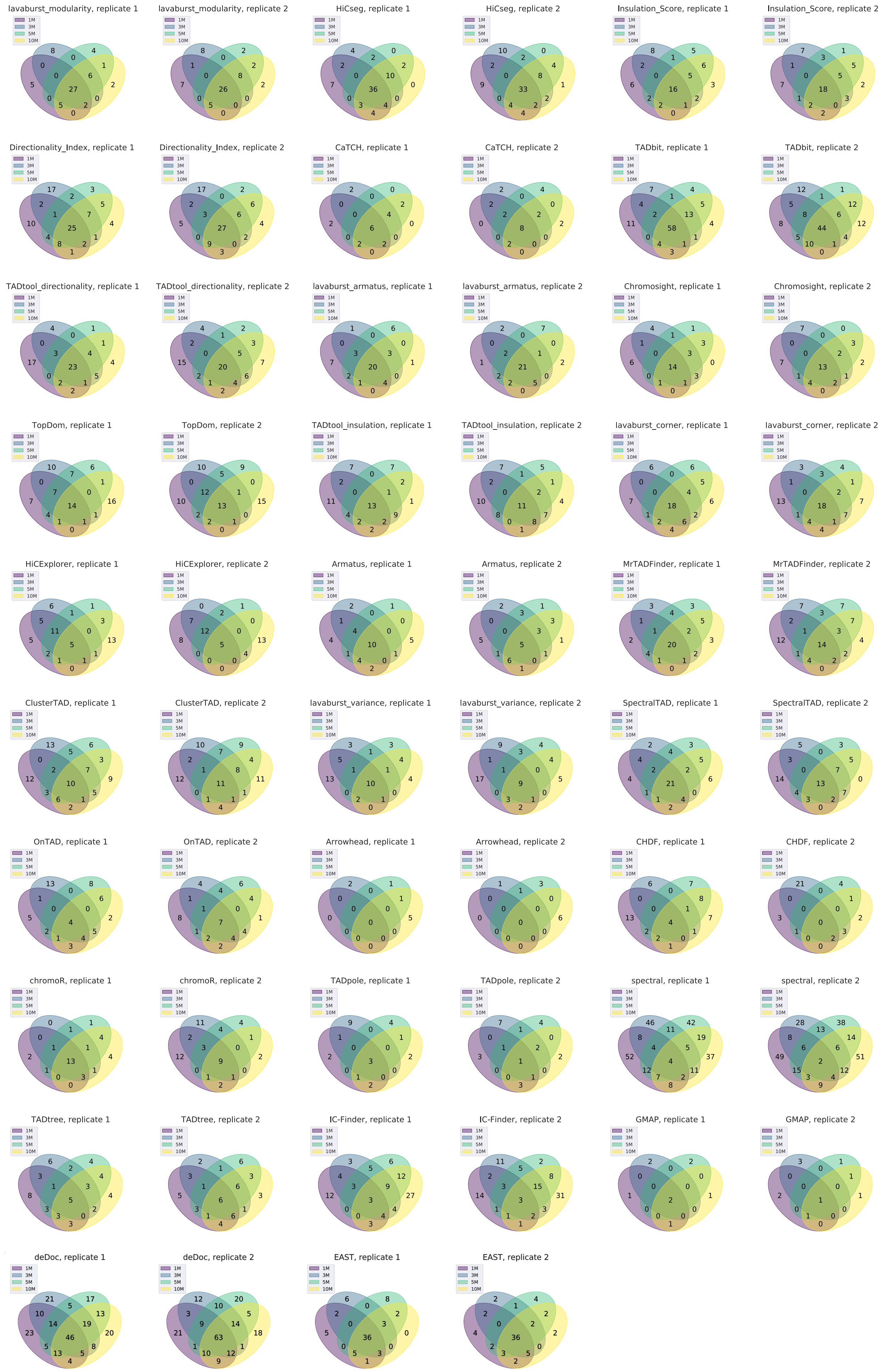

Supplementary Figure S8. Venn diagrams showing the number of intersections between the CID boundaries annotated by each domain caller for *E.coli* pseudo-replicates with varying coverage (1, 3, 5 and 10 millions of contacts).

Supplementary Figure S9

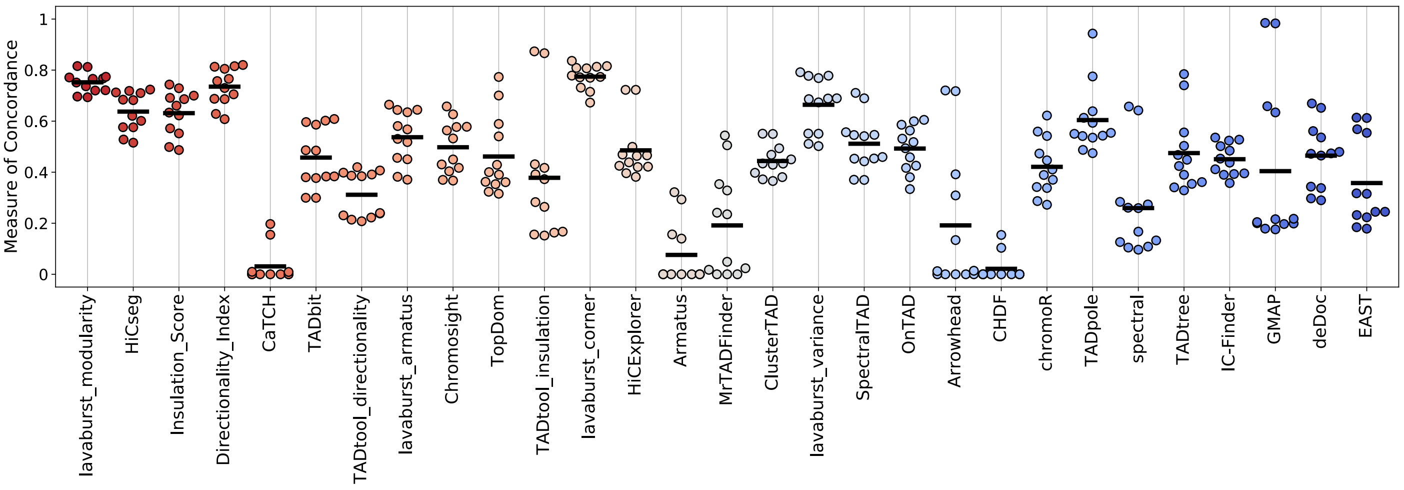

Supplementary Figure S9. Swarm plots for the Measure of Concordance values between the CID segmentations annotated for *E. coli* pseudo-replicate contact maps with different resolutions (3, 5, 10 and 15 kb) by each domain caller. Each dot represents a comparison between CID annotations obtained for two maps with different resolutions. The black line represents the median MoC value for each tool.

Supplementary Figure S10

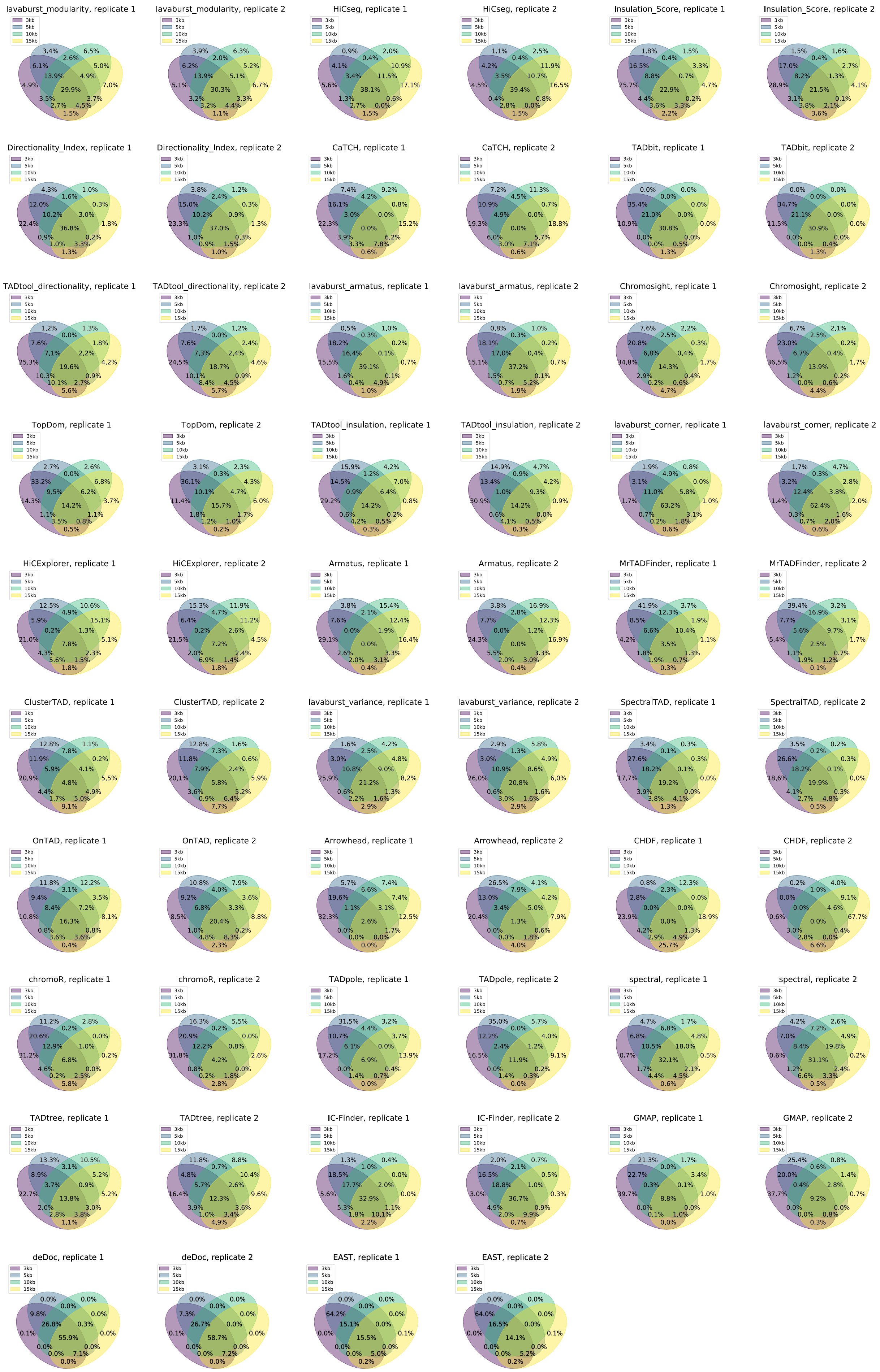

Supplementary Figure S10. Venn diagrams showing the percentage of overlap between the “extended” CID boundaries annotated by each domain caller for *E. coli* pseudo-replicates with different resolutions (3, 5, 10 and 15 kb).

Supplementary Figure S11

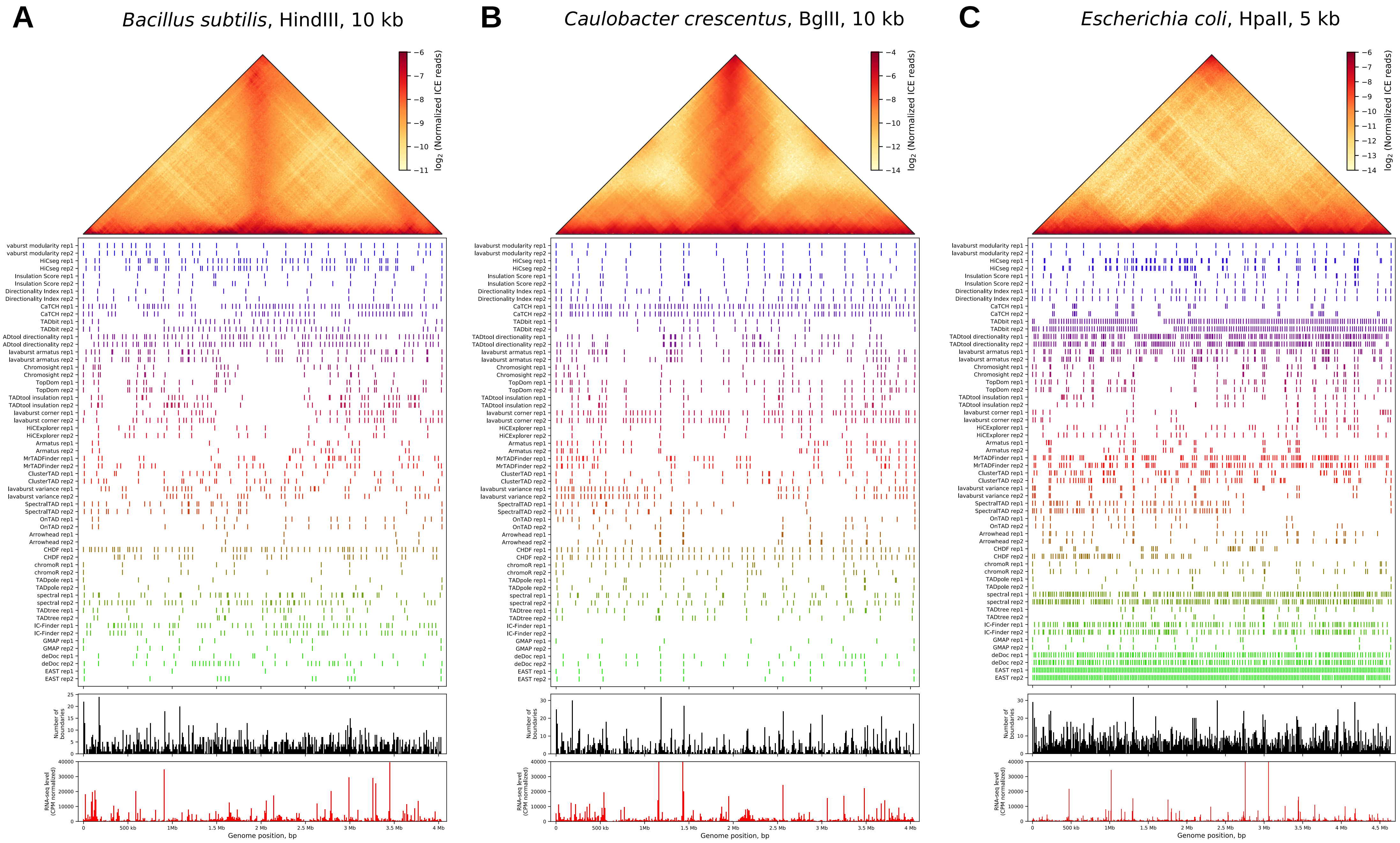

Supplementary Figure S11. The association between the annotated domain boundaries and gene expression along the genome. From top to bottom: (i) normalized Hi-C contact map, (ii) a pile up of the domain boundaries predicted by each domain calling tool for both replicates, (iii) a consensus of these boundaries and (iv) gene expression track for (A) *C. crescentus*, (B) *B. subtilis* and (C) *E. coli*.

Supplementary Figure S12

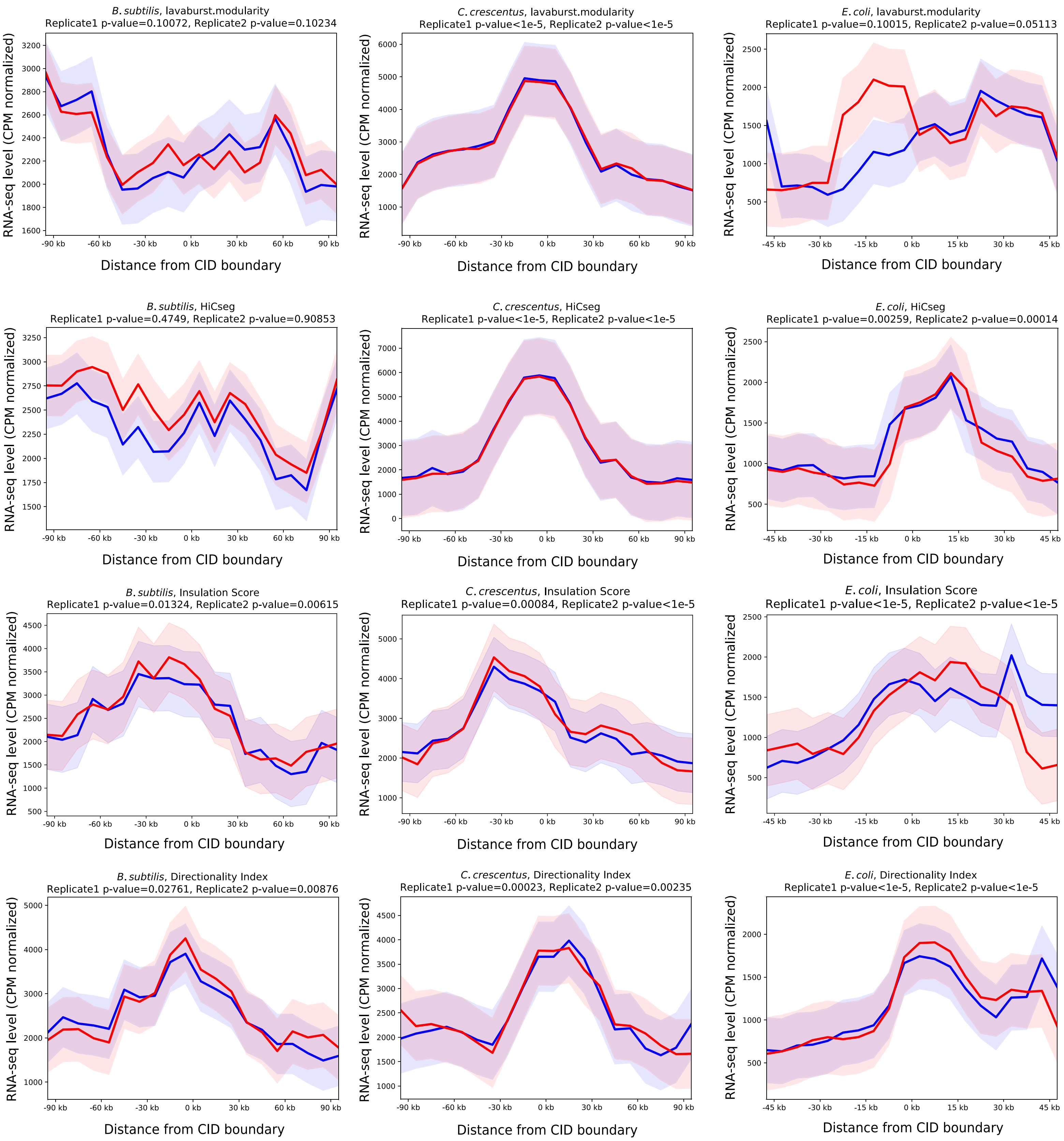

Supplementary Figure S12. Gene expression levels at the CID boundaries annotated by lavaburst.modularity, HiCseg, Insulation Score and Directionality Index in *B. subtilis*, *C. crescentus* and *E. coli* datasets. Lines show the mean normalized RNA-seq expression levels in the vicinity of CID boundaries (the profile around boundaries annotated in replicate 1 are in blue, and those annotated in replicate 2 are in red) with a shaded area indicating the range of one standard deviation from the mean expression. The p-values for significance of the expression difference at the boundaries compared to the regions within CIDs are given in the subplots' titles.

Supplementary Table S1

Organism	Hi-C datasets	RNA-seq datasets	Restriction enzyme	Genome	Resolution	Used for domain-calling tools benchmark
Bacillus subtilis	rep1 - SRR2002580<br>rep2 - SRR2002581	SRR922367	HindIII	NC_022898.1	10 kb	Yes
	rep1 - SRR2182094		HpaII		5 kb	No
Caulobacter crescentus	rep1 - SRR824843<br>rep2 - SRR824844	SRR2818123	BglII	NC_011916.1	10 kb	Yes
	rep1 - SRR824846		NcoI			No
Escherichia coli	rep1 - SRR6354545, SRR6940991, SRR6940992, SRR6843204, SRR6843203<br>rep2 - SRR6354546	SRR2932231	HpaII	NC_000913.3	5 kb	Yes
Mycoplasma pneumoniae	rep1 - ERR1413600<br>rep2 - ERR1413595<br>rep3 - ERR1413596<br>rep4 - ERR1413597<br>rep5 - ERR1413598	-	HpaII	NC_000912.1	3 kb	Yes
	rep1 - ERR1413593<br>rep2 - ERR1413594		HindIII			
Sulfolobus acidocaldarius	rep1 - SRR8699887<br>rep2 - SRR8699888<br>rep3 - SRR8699889	-	HindIII	CP000077.1	15 kb	Yes

Supplementary Table S1. Accession numbers of the publicly available Hi-C, 3C-seq and RNA-seq datasets (in Sequence Read Archive or European Nucleotide Archive) and reference genomes (in Genbank) used in the study.

Supplementary Table S2

Domain caller	Parameters selection	Reference
**Armatus**	We used Armatus v. 2.1 and varied the *gamma* parameter from 0.01 to 5.00 with a step of 0.01 to find the optimal CIDs segmentation of a genome.	[1]
**Arrowhead**	The Juicer Tools v. 1.11.09 arrowhead module with default parameters was used to determine CIDs. The nested domains were then discarded to remove the hierarchy.	[2]
**CaTCH**	From the CaTCH package we used the *domain.call* function with no parameters to annotate CIDs.	[3]
**CHDF**	We executed CHDF with parameters *Length*, *Number* and *Size* equal to the dimensions of the corresponding contact map.	[4]
**chromoR**	The function *segmentCIM* from the chromoR was used on the raw contact maps to find the domains.	[5]
**Chromosight**	The Chromosight package was used in the detect mode with parameter *pattern* = 'borders'.	[6]
**ClusterTAD**	We used Java implementation of ClusterTAD with the parameters *window* set to 10 and minimum CID size set to 4 bins. The search for optimal CID boundaries was performed among CID sets identified with different *K* values used for clustering.	[7]
**deDoc**	We used deDoc(M) algorithm implementation with default parameters to identify CIDs.	[8]
**Directionality_Index**	We used the Directionality Index approach based on paired t-test. For the matrices with resolution higher than 10 kb, the number of bins considered for t-statistics calculation was set to 20, while for the lower resolution matrices it was set to 10. We then manually annotated CID boundaries using the directionality index profile and t-statistics threshold at significance level 0.1.	[9]
**EAST**	The EAST script was executed with parameters *maxW* = 50 and *minL* = 4.	[10]
**GMAP**	GMAP was executed on the raw contact maps with parameters *dom_order* set to 1, *min_dp* set to 4, *max_dp* set to 100, *min_d* set to 10 and *fcthr* set to 0.75.	[11]
**HiCExplorer**	To identify CIDs with HiCExplorer, we used its hicFindTADs module with *minDepth* parameter ranging from 3 to 20 matrix resolutions and *delta* from 0.005 to 0.1 with a step of 0.005. A correction for multiple testing was performed using the FDR method.	[12]
**HiCseg**	The HiCseg was executed with parameters *distrib* = "G" and *model* = "D". We varied the *nb_change_max* parameter from 2 to 100 to find the optimal domains.	[13]
**IC-Finder**	In order to identify CIDs, IC-Finder was executed with default parameters.	[14]
**Insulation_Score**	We used the original Perl implementation of the Insulation Score algorithm and varied the *IS* parameter from 5 to 15 resolution sizes with a step of 1 and the *IDS* parameter from 2 to 10 resolution sizes with a step of 2 in order to find the optimal segmentation of CIDs.	[15]
**lava.armatus**	The Lavaburst package (https://github.com/nvictus/lavaburst) includes four domain calling algorithms: armatus, corner, modularity and variance. For each algorithm, we varied the *gamma* parameter in the range from 0.01 to 5.00 with a step of 0.01 to find the optimal CIDs.	
**lava.corner**		
**lava.modularity**		
**lava.variance**		
**MrTADFinder**	We used MrTADFinder v. 1.2 and varied the *res* parameter from 0.5 to 3.5 with a step of 0.1 to find the optimal segmentation of CIDs.	[16]
**OnTAD**	We used OnTAD with parameter *minsz* set to 4. For *C.crescentus*, *B.subtilis* and *E.coli* we then merged the annotated domains at levels 2, 3 and 4 into one set.	[17]
**spectral**	For spectral algorithm we varied the *lambda* threshold parameter from 0.01 to 1.00 with a step of 0.01 to find the optimal CIDs annotation.	[18]
**SpectralTAD**	SpectralTAD was executed using a raw contact map as input data and a minimum CID size of 4 bins.	[19]
**TADbit**	We used a raw contact map as input for TADbit and set the *resolution* parameter to correspond to each matrix.	[20]
**TADpole**	We used TADpole with parameter *resol* corresponding to each matrix size.	[21]
**TADtool.directionality**	We used directionality index and insulation index algorithms from the TADtool. We varied the *cutoff* parameter from 0 to 0.02 with a step of 0.0001 and the *window_size* parameter from 20000 to 200000 with a step of 10000 in order to find the optimal CIDs.	[22]
**TADtool.insulation**		
**TADtree**	In order to obtain domain annotation using TADtree, we set the *S* to 50 and the *N* to 100. The domains annotated at all N values were merged and nested domains were then discarded to remove the hierarchy.	[23]
**TopDom**	For TopDom, we varied the *window_size* parameter from 2 to 20 with a step of 1 to find the optimal segmentation.	[24]

Supplementary Table S2. The description of the parameters for the domain calling procedure.

Supplementary Table S3

Domain caller	*C. crescentus*	*B. subtilis*	*E. coli*	*M. pneumoniae* HindIII	*M. pneumoniae* HpaII	*S.acidocaldarius*
**Armatus**	gamma = 0.16	gamma = 0.01	gamma = 0.07	gamma = 0.09	gamma = 0.12	gamma = 0.01
**Arrowhead**	-	-	-	-	-	-
**CaTCH**	-	-	-	-	-	-
**CHDF**	-	-	-	-	-	-
**chromoR**	-	-	-	-	-	-
**Chromosight**	-	-	-	-	-	-
**ClusterTAD**	K = 22	K = 19	K = 31	K = 5	K = 3	K = 16
**deDoc**	-	-	-	-	-	-
**Directionality_Index**	-	-	-	-	-	-
**EAST**	-	-	-	-	-	-
**GMAP**	-	-	-	-	-	-
**HiCExplorer**	window_size = 110000<br>cutoff = 0.03	window_size = 70000<br>cutoff = 0.005	window_size = 25000<br>cutoff = 0.005	window_size = 45000<br>cutoff = 0.01	-	window_size = 90000<br>cutoff = 0.01
**HiCseg**	Kmax = 14	Kmax = 75	Kmax = 79	Kmax = 19	Kmax = 21	Kmax = 16
**IC-Finder**	-	-	-	-	-	-
**Insulation_Score**	IS = 130000<br>IDS = 20000	IS = 110000<br>IDS = 60000	IS = 75000<br>IDS = 10000	IS = 15000<br>IDS = 30000	IS = 18000<br>IDS = 12000	IS = 90000<br>IDS = 60000
**lava.armatus**	gamma = 0.66	gamma = 1.12	gamma = 0.78	gamma = 1.33	gamma = 1.13	gamma = 0.08
**lava.corner**	gamma = 0.81	gamma = 0.95	gamma = 1.15	gamma = 1.01	gamma = 0.99	gamma = 0.65
**lava.modularity**	gamma = 2.81	gamma = 2.16	gamma = 2.36	gamma = 1.28	gamma = 1.62	gamma = 1.43
**lava.variance**	gamma = 1.36	gamma = 0.51	gamma = 0.87	gamma = 0.58	gamma = 0.61	gamma = 0.73
**MrTADFinder**	res = 1.2	res = 1.0	res = 0.8	res = 1.1	res = 1.0	res = 1.0
**OnTAD**	-	-	-	-	-	-
**spectral**	lambda = 0.64	lambda = 0.74	lambda = 0.49	lambda = 0.87	lambda = 0.69	lambda = 0.58
**SpectralTAD**	-	-	-	-	-	-
**TADbit**	-	-	-	-	-	-
**TADpole**	level = 11	level = 5	level = 6	level = 15	level = 1	level = 5
**TADtool.directionality**	window_size = 20000<br>cutoff = 0.0095	window_size = 40000<br>cutoff = 0.0	window_size = 30000<br>cutoff = 0.0	window_size = 6000<br>cutoff = 0.0	window_size = 51000<br>cutoff = 0.0	window_size = 150000<br>cutoff = 0.0
**TADtool.insulation**	window_size = 70000<br>cutoff = 0.0148	window_size = 20000<br>cutoff = 0.0097	window_size = 75000<br>cutoff = 0.0066	window_size = 12000<br>cutoff = 0.0047	window_size = 6000<br>cutoff = 0.0046	window_size = 30000<br>cutoff = 0.0183
**TADtree**	-	-	-	-	-	-
**TopDom**	window_size = 7	window_size = 8	window_size = 14	window_size = 6	window_size = 5	window_size = 8

Supplementary Table S3. Optimal run parameters of the domain callers for each dataset as revealed by the Jaccard Index maximization analysis.

Supplementary Table S4

Domain caller	Reproducibility between replicates		Robustness to resolution and/or coverage			Concordance with other domain calling tools			Enrichment of biological features at the domain boundaries			
	Magnitov *et al.*	Forcato *et al.*	Magnitov *et al.*	Dali and Blanchett	Zufferey *et al.*	Magnitov *et al.*	Dali and Blanchett	Zufferey *et al.*	Magnitov *et al.*	Dali and Blanchett	Forcato *et al.*	Zufferey *et al.*
**Armatus**			no					no		yes	yes	no
**Arrowhead**		no	no	no		yes		yes		yes	yes	yes
**CaTCH**	yes	N/A	no	N/A	yes	no	N/A	yes		N/A	N/A	
**CHDF**		N/A	no	N/A	yes		N/A	yes		N/A	N/A	yes
**chromoR**		N/A		N/A			N/A			N/A	N/A	
**Chromosight**		N/A	yes	N/A	N/A		N/A	N/A		N/A	N/A	N/A
**ClusterTAD**		N/A		N/A			N/A	no		N/A	N/A	no
**deDoc**	no	N/A		N/A	N/A		N/A	N/A		N/A	N/A	N/A
**Directionality Index**	yes	no	yes	yes			yes	yes	yes	yes	yes	yes
**EAST**	no	N/A	yes	N/A		no	N/A			N/A	N/A	
**GMAP**	no	N/A	yes	N/A		no	N/A	yes		N/A	N/A	yes
**HiCExplorer**		N/A		N/A			N/A			N/A	N/A	
**HiCseg**	yes	yes	yes	yes	yes		yes	yes	no	yes	yes	yes
**IC-Finder**	no	N/A		N/A	yes	no	N/A	yes		N/A	N/A	yes
**Insulation Score**	yes		yes	N/A	yes		N/A	no	yes	N/A	yes	
**lava.armatus**		N/A	yes	N/A	N/A		N/A	N/A		N/A	N/A	N/A
**lava.corner**		N/A	yes	N/A	N/A		N/A	N/A		N/A	N/A	N/A
**lava.modularity**	yes	N/A	yes	N/A	N/A		N/A	N/A	no	N/A	N/A	N/A
**lava.variance**		N/A	yes	N/A	N/A	no	N/A	N/A		N/A	N/A	N/A
**MrTADFinder**		N/A	no	N/A			N/A			N/A	N/A	
**OnTAD**		N/A		N/A	N/A	yes	N/A	N/A		N/A	N/A	N/A
**spectral**	no	N/A	no	N/A		no	N/A	no		N/A	N/A	no
**SpectralTAD**		N/A	yes	N/A	N/A		N/A	N/A		N/A	N/A	N/A
**TADbit**	yes		yes		yes	no		yes	no	yes	yes	
**TADpole**	no	N/A		N/A	N/A	yes	N/A	N/A		N/A	N/A	N/A
**TADtool.directionality**	yes	N/A		N/A	N/A		N/A	N/A		N/A	N/A	N/A
**TADtool.insulation**		N/A		N/A	N/A		N/A	N/A		N/A	N/A	N/A
**TADtree**	no		yes							yes	yes	
**TopDom**		N/A		yes	yes	yes	yes	yes	yes	yes	N/A	yes

Supplementary Table S4. The comparative analysis of the results from this work and previously published benchmarks. N/A means the tool was not analysed in the corresponding study.

References

1. Filippova,D. *et al*. (2014) Identification of alternative topological domains in chromatin. *Algorithms Mol. Biol.*, **9**, 14.
2. Durand,N.C. *et al*. (2016) Juicer Provides a One-Click System for Analyzing Loop-Resolution Hi-C Experiments. *Cell Syst*, **3**, 95–98.
3. Zhan,Y. *et al*. (2017) Reciprocal insulation analysis of Hi-C data shows that TADs represent a functionally but not structurally privileged scale in the hierarchical folding of chromosomes. *Genome Res.*, **27**, 479–490.
4. Wang,Y. *et al*. (2015) A novel method to identify topological domains using Hi-C data. *Quant Biol*, **3**, 81–89.
5. Shavit,Y. and Lio',P. (2014) Combining a wavelet change point and the Bayes factor for analysing chromosomal interaction data. *Mol. Biosyst.*, **10**, 1576–1585.
6. Matthey-Doret,C. *et al*. (2020) Chromosight: A computer vision program for pattern detection in chromosome contact maps. *bioRxiv*, 2020.03.08.981910.
7. Oluwadare,O. and Cheng,J. (2017) ClusterTAD: an unsupervised machine learning approach to detecting topologically associated domains of chromosomes from Hi-C data. *BMC Bioinformatics*, **18**, 1–14.
8. Li,A. *et al*. (2018) Decoding topologically associating domains with ultra-low resolution Hi-C data by graph structural entropy. *Nat. Commun.*, **9**, 3265.
9. Le,T.B.K. *et al*. (2013) High-resolution mapping of the spatial organization of a bacterial chromosome. *Science*, **342**, 731–734.
10. Roayaei Ardakany,A. and Lonardi,S. (2017) Efficient and Accurate Detection of Topologically Associating Domains from Contact Maps. *17th International Workshop on Algorithms in Bioinformatics (WABI 2017)*, **88**, 22:1–22:11.
11. Yu,W. *et al*. (2017) Identifying topologically associating domains and subdomains by Gaussian Mixture model And Proportion test. *Nat. Commun.*, **8**, 535.
12. Ramírez,F. *et al*. (2018) High-resolution TADs reveal DNA sequences underlying genome organization in flies. *Nat. Commun.*, **9**, 189.
13. Lévy-Leduc,C. *et al*. (2014) Two-dimensional segmentation for analyzing Hi-C data. *Bioinformatics*, **30**, i386–i392.
14. Haddad,N. *et al*. (2017) IC-Finder: inferring robustly the hierarchical organization of chromatin folding. *Nucleic Acids Res.*, **45**, e81.
15. Crane,E. *et al*. (2015) Condensin-driven remodelling of X chromosome topology during dosage compensation. *Nature*, **523**, 240–244.
16. Yan,K.-K. *et al*. (2017) MrTADFinder: A network modularity based approach to identify topologically associating domains in multiple resolutions. *PLoS Comput. Biol.*, **13**, e1005647.
17. An,L. *et al*. (2019) OnTAD: hierarchical domain structure reveals the divergence of activity among TADs and boundaries. *Genome Biol.*, **20**, 282.
18. Chen,J. *et al*. (2016) Spectral identification of topological domains. *Bioinformatics*, **32**, 2151–2158.
19. Cresswell,K.G. *et al*. (2019) SpectralTAD: an R package for defining a hierarchy of Topologically Associated Domains using spectral clustering. *bioRxiv*, 549170.
20. Serra,F. *et al*. (2017) Automatic analysis and 3D-modelling of Hi-C data using TADbit reveals structural features of the fly chromatin colors. *PLoS Comput. Biol.*, **13**, e1005665.
21. Soler-Vila,P. *et al*. (2020) Hierarchical chromatin organization detected by TADpole. *Nucleic Acids Res.*, **48**, e39.
22. Kruse,K. *et al*. (2016) TADtool: visual parameter identification for TAD-calling algorithms. *Bioinformatics*, **32**, 3190–3192.
23. Weinreb,C. and Raphael,B.J. (2016) Identification of hierarchical chromatin domains. *Bioinformatics*, **32**, 1601–1609.
24. Shin,H. *et al*. (2016) TopDom: an efficient and deterministic method for identifying topological domains in genomes. *Nucleic Acids Res.*, **44**, e70–e70.
